# Discriminative validity of the lower and upper quarter Y balance test performance: a comparison between healthy trained and untrained youth

**DOI:** 10.1186/s13102-020-00220-w

**Published:** 2020-12-03

**Authors:** Gerrit Schwiertz, Rainer Beurskens, Thomas Muehlbauer

**Affiliations:** 1 Division of Movement and Training Sciences/Biomechanics of Sport, University of Duisburg-Essen, Gladbecker Str. 182, 45141 Essen, Germany; 2 Department of Health and Social Affairs, FHM Bielefeld - University of Applied Sciences, Bielefeld, Germany

**Keywords:** Postural control, Shoulder mobility/stability, Young athletes, Validation

## Abstract

**Background:**

The Lower (YBT-LQ) and Upper (YBT-UQ) Quarter Y Balance Test have been widely used for the assessment of dynamic balance and shoulder mobility/stability, respectively. However, investigations on the validity of the two tests in youth are lacking. Therefore, we performed two studies to determine discriminative validity of the YBT-LQ (study 1) and the YBT-UQ (study 2) in healthy youth.

**Methods:**

Sixty-nine male soccer players (age: 14.4 ± 1.9 yrs) and 69 age-matched untrained male subjects (14.3 ± 1.6 yrs) participated in study 1 and 37 young swimmers (age: 12.3 ± 2.1 yrs) as well as 37 age−/sex-matched individuals (age: 12.5 ± 2.0 yrs) took part in study 2. Absolute (cm) and relative (% leg/arm length) maximal reach distances per reach direction and the composite score of the YBT-LQ/UQ were used as outcome measures. One-way analysis of variance and the receiver operator characteristic curve analysis (i.e., calculating the area under the curve [AUC]) were conducted to assess discriminative validity.

**Results:**

Concerning the relative values, youth athletes showed significantly better YBT-LQ (study 1: *p* < 0.001, *d* = 0.86–1.21) and YBT-UQ (study 2: *p* < 0.001, *d* = 0.88–1.48) test performances compared to age- and sex-matched untrained subjects. Further, AUC-values indicated a chance of ≥74% (YBT-LQ) and ≥ 71% (YBT-UQ) to discriminate between youth athletes and controls. These findings were confirmed when using the absolute data for analysis.

**Conclusions:**

According to our results, the YBT-LQ and the YBT-UQ seem to be useful test instruments to discriminate trained and untrained healthy youth performance for dynamic balance and shoulder mobility/stability, respectively.

## Background

The Y-Balance-Test (YBT) represents a field-based test [1, 2] assessing i) dynamic balance performance when applied to lower extremities (YBT-LQ) [3] and ii) shoulder mobility/stability when applied to upper extremities (YBT-UQ) [4]. Both testing procedures have widely been used in previous literature to determine influencing factors such as age [5, 6], sex [6, 7], anthropometric characteristics [8, 9], limb dominance/strength [4, 10], and previous injuries [11, 12] and to detect performance differences in different cohorts [3, 13–15].

Concerning the latter aspect, Butler et al. [3] investigated YBT-LQ performance in male high school, collegiate, and professional soccer players and detected significantly lower reach distances in the posteromedial and posterolateral directions for the high school players than the two other groups of player. Further, Bullock et al. [13] studied middle school, high school, college, and professional basketball players and observed that high school players performed significantly better (i.e., anterior reach direction) compared to middle school and college players. Regarding the YBT-UQ, Bullock et al. [14] tested high school and collegiate swimmers and found significantly better values for the medial reach direction in favour of the latter group of swimmers. In another study, Krysak et al. [15] compared middle school, high school, college, and professional golfer players and reported greater reach distances in the medial, inferolateral, and superolateral directions for the professional golfers compared to the three other groups.

Results of the aforementioned studies shed light onto performance differences based on the athletes’ level of competition and experience. However, not only their level of competition but also the age differed between cohorts. For example, in the study conducted by Butler and colleagues [3], age differed between 15.6 years in high school soccer players and 26.2 years in professional soccer players. Further, participants in Krysak and co-workers [15] were aged between 12.2 years (middle school golfers) und 31.8 years (professional golfers). Thus, participants’ age might have influenced performance differences in these studies and it remains unclear whether the YBT-LQ/UQ is discriminatively valid. As a consequence, studies comparing age- and sex-matched persons with various levels of competition are needed.

Most notably, examining discriminative validity of the YBT-LQ/UQ in children and adolescents is important since these age groups are used for talent selection and scouting [16]. More precisely, the investigation of age−/sex-matched youth in relation to their training status is useful to discriminate high-performer versus low-performer using the YBT-LQ/UQ. Thus, the aim of the present study was to determine discriminative validity of the YBT-LQ (study 1) and the YBT-UQ (study 2) by comparing age- and sex-matched trained versus untrained youth. With reference to the relevant literature [3, 13–15], we expected better performances in both tests for trained compared to untrained youth and we hypothesized good discriminative validity for both tests.

## Methods

### Participants

Participants’ characteristics are summarized in Table 1. In study 1, 69 male soccer players from a local sports club and 69 age-matched untrained male subjects performed the YBT-LQ. In study 2, 37 female and male swimmers from a local sports club and 37 age−/sex-matched untrained individuals conducted the YBT-UQ. The maturity offset was calculated in terms of years from peak height velocity (PHV) for each participant by using the formula provided by Moore et al. [17]. Participants’ assent and parents’ written informed consent were obtained prior to the start of the study. The Human Ethics Committee at the University of Duisburg-Essen, Faculty of Educational Sciences approved the study protocol.

**Table 1:** Characteristics of the participants by study

Characteristic	Study 1 (*N* = 138)			Study 2 (*N* = 74)		
	Soccer players (*n* = 69)	Controls (*n* = 69)	*p*-value	Swimmers (*n* = 37)	Controls (*n* = 37)	*p*-value
Age (yrs)	14.4 ± 1.9	14.3 ± 1.6	.511	12.3 ± 2.1	12.5 ± 2.0	.650
Sex (f/m)	0/69	0/69		22/15	22/15	
Body mass (kg)	60.9 ± 14.8	64.8 ± 15.8	.136	49.2 ± 14.8	52.6 ± 17.8	.378
Body height (cm)	169.6 ± 12.5	173.5 ± 12.4	.069	160.8 ± 14.7	160.5 ± 14.7	.928
BMI (kg/m^2^)	20.8 ± 2.9	21.3 ± 4.0	.419	18.6 ± 2.5	19.8 ± 3.3	.079
Maturity offset (yrs from PHV)	1.0 ± 1.5	0.9 ± 2.7	.750	0.1 ± 2.0	0.3 ± 1.9	.618
Left leg length (cm)	90.2 ± 6.2	94.1 ± 7.7	.196	–	–	–
Right leg length (cm)	90.1 ± 6.1	94.0 ± 7.5	.184	–	–	–
Left arm length (cm)	–	–	–	81.8 ± 8.7	81.0 ± 8.2	.686
Right arm length (cm)	–	–	–	81.9 ± 8.8	81.2 ± 8.3	.706
Trunk length (cm)	79.8 ± 8.3	80.2 ± 8.2	.419	–	–	–
Relative lower limb length (%)	53.1 ± 2.0	54.0 ± 2.8	.504	–	–	–

Data are mean ± standard deviation

*BMI* body mass index, *f* female, *m* male, *PHV* peak height velocity

### Testing procedures

Discriminative validity of the YBT-LQ and the YBT-UQ was assessed in study 1 and study 2, respectively. In both studies, we used a standardized general warm-up comprising 5 min of running at a moderate speed and a test-specific warm-up consisting of three submaximal reaches per arm/leg and reach direction. All participants received standardized verbal instructions and a visual demonstration regarding the testing procedure that included assessment of anthropometric variables (i.e., body mass, body height, arm length [AL], leg length [LL]) followed by performance assessment in the YBT-LQ (study 1) or YBT-UQ (study 2). The participants had no prior experience with the YBT-LQ/UQ.

#### Assessment of anthropometric variables

Body mass (kg) was measured in light clothing and without shoes to the nearest 100 g with an electronic scale (seca 803, Basel, Switzerland). Further, body height (cm) was determined without shoes to the nearest 0.5 cm with a stadiometer (seca 217, Basel, Switzerland). Body mass index was calculated using body mass divided by height squared (kg/m^2^). Length (cm) of the right and left arm was determined with a cloth tape measure from the seventh cervical spinous process to the distal tip of the middle finger with the shoulder being in a 90° abduction [18]. Further, left and right leg length (cm) were assessed by measuring the distance from the anterior superior iliac spine to the most distal aspect of the medial malleolus using a cloth tape with the participant lying supine [19]. In accordance to Fusco et al. [8], trunk length was calculated as the difference between body height and LL and relative lower limb length was determined using LL divided by body height and then multiplied by 100.

#### Assessment of lower quarter Y balance test performance

**Fig. 1:**
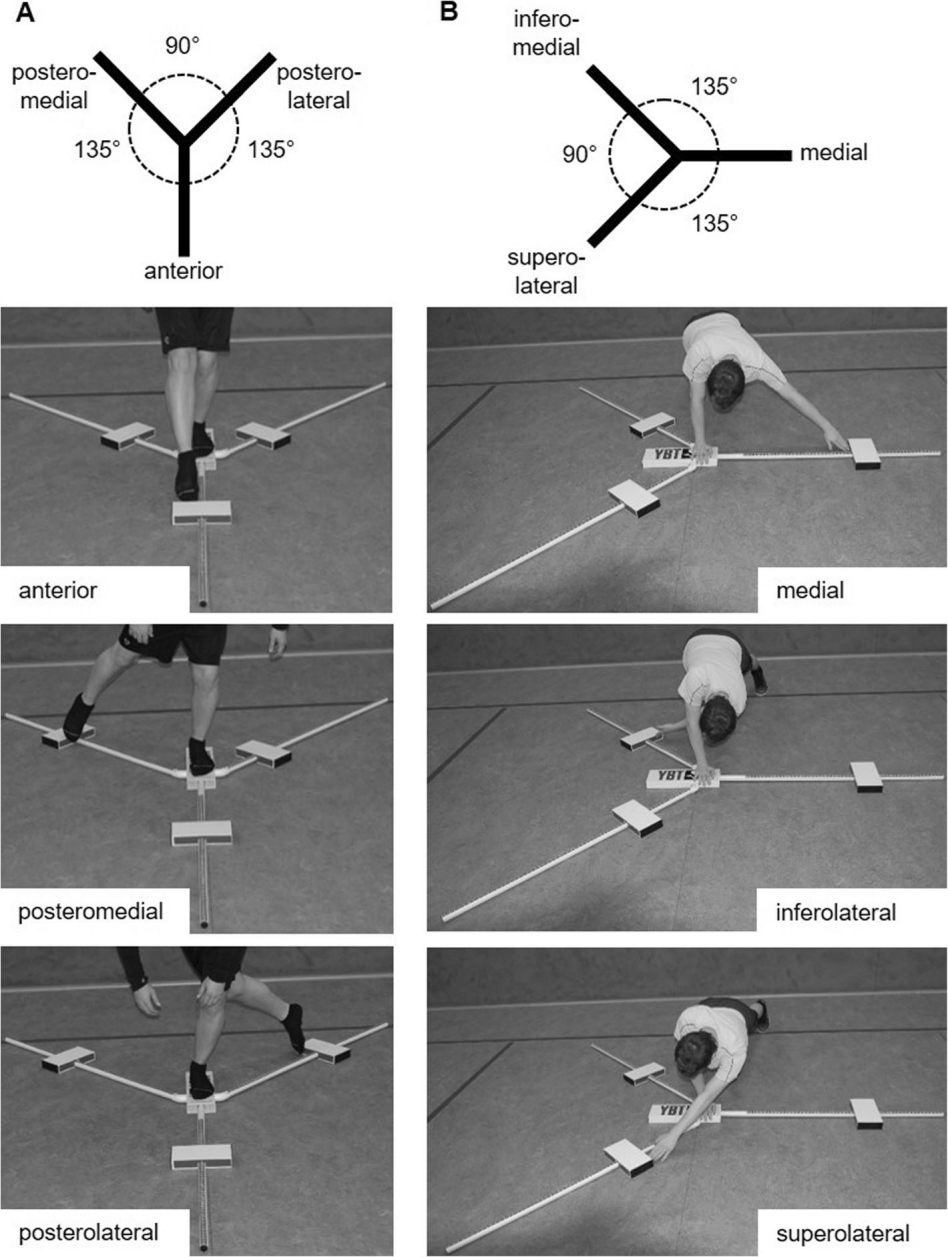
Setup for the assessment of Lower (**a**) and Upper (**b**) Quarter Y Balance Test performance

YBT-LQ performance was assessed by means of the YBT Kit (Functional Movement Systems®, Chatham, USA). The test kit consists of a centralized platform to which three pipes were attached representing the anterior (AT), posteromedial (PM), and posterolateral (PL) reach directions (Fig. 1a). Each pipe is marked in 1.0-cm increments for measurement purposes and equipped with a moveable reach indicator. The participants were asked to move the reach indicator as far as possible into the AT direction with the right leg while standing on the centralized platform with their left leg followed by standing on the right leg and reaching with the left leg. This protocol was then replicated for the PM and PL directions. Each participant performed three practice trials followed by three data-collection trials per leg and reach direction. A one-minute rest was provided between trials. The absolute maximal reach distance (cm) per leg and reach direction was used for further analysis. Reliability of the YBT-LQ has been shown to be predominately “excellent” in healthy youth [20].

#### Assessment of upper quarter Y balance test performance

The YBT Kit was also used for the assessment of YBT-UQ performance, with the three pipes representing the medial (MD), inferolateral (IL), and superolateral (SL) reach directions (Fig. 1b). Participants were instructed to move the reach indicator with the right arm as far as possible in the MD, IL, and SL directions while maintaining a weight bearing one-arm push-up position with their left arm on the centralized platform. This protocol was then replicated for the left arm. Three practice trials were conducted followed by three data-collection trials. The rest between trials comprised 1 min. The best values (i.e., absolute maximal reach distance in cm) per arm and reach direction was used for further analysis. The reliability of the YBT-UQ ranged from “moderate-to-good” to “excellent” in healthy youth [21].

### Data and statistical analyses

For both tests, relative/normalized maximal reach distances (%) per reach direction and leg/arm were calculated by dividing the absolute maximal reach distance (cm) by LL or AL (cm) and then multiplying by 100. In addition, the normalized (%) composite score (CS) per leg/arm was computed as the sum of the absolute maximal reach distance (cm) per reach direction divided by three times LL or AL and then multiplied by 100.

Further, the mean value was calculated as a measure of central tendency and the standard deviation (SD) as a dispersion measure. Normal distribution was examined using the Shapiro-Wilk test (*p* > 0.05) and homogeneity of variances using the Levene test (*p* > 0.05). An independent samples t-test was used to quantify differences between the cohorts. Discriminative validity was analyzed using the one-way analysis of variance (ANOVA). Statistically significant differences were identified at *p* < 0.05. Furthermore, effect size (Cohen’s *d*) was calculated and classified as “small” (0 ≤ *d* ≤ 0.49), “moderate” (0.50 ≤ *d* ≤ 0.79), and “large” (*d* ≥ 0.80) [22]. Moreover, we conducted a receiver operator characteristic (ROC) curve analysis and calculated the area under the receiver operator characteristic (AUC) curve for each outcome measure (i.e., per reach direction and composite score) separately. The AUC measures the entire two-dimensional area underneath the entire ROC curve. In this regard, Deyo and Centor [23] stated that an AUC-value of 0.50 indicates “no “and an AUC-value of 1.0 indicates “perfect” discriminative validity. All statistical analyses were performed using Statistical Package for Social Sciences version 24.0 (SPSS Inc., Chicago, IL, USA).

## Results

### Characteristics of the study participants

Irrespective of outcome, we did not detect statistically significant differences in participants’ characteristics, neither in study 1 nor in study 2 (Table 1).

### Discriminative validity of lower quarter Y balance test performance (study 1)

Statistical data on the discriminative validity for YBT-LQ performance between young male soccer players and age-matched male untrained subjects are displayed in Table 2. With regard to the absolute values (i.e., reach distance in cm), the soccer players compared to the controls achieved small- to medium-sized and significantly better values for AT (*p* ≤ 0.009; *d* = 0.45–0.49), PM (*p* < 0.001; *d* = 0.64), and PL (*p* < 0.001; *d* = 0.65–0.69) directions, irrespective of the reaching leg. In addition, we detected significant differences between the three reach directions (PM > PL > AT) for the right and left leg reach, irrespective of training status (i.e., soccer players or untrained subjects). Further, AUC-values were 0.63 (AT), 0.67 (PM), and 0.69 (PL) for the right and 0.64 (AT), 0.66 (PM), and 0.68 (PL) for the left leg reach, respectively. Results indicate that there is a chance of 63–69% that the YBT-LQ is possible to differentiate between soccer players and age-matched untrained individuals.

**Table 2:** Discriminative validity for the assessment of absolute (cm) and relative (% leg length) Lower Quarter Y Balance Test performance between trained (i.e., soccer players) and age-matched untrained (i.e., controls) youth

	Participants (*N* = 138)		Statistics	
	Soccer players (*n* = 69)	Controls (*n* = 69)	*p*-value (*d*)	AUC-value
*Right leg reach*				
AT (cm)	70.9 ± 7.6	67.2 ± 7.4	.004 (0.49)	.63
PM (cm)	107.6 ± 8.5	101.1 ± 11.4	<.001 (0.64)	.67
PL (cm)	103.9 ± 7.8	97.7 ± 10.2	<.001 (0.69)	.69
*Left leg reach*				
AT (cm)	69.6 ± 7.5	66.0 ± 8.2	.009 (0.45)	.64
PM (cm)	106.5 ± 8.2	99.5 ± 13.1	<.001 (0.64)	.66
PL (cm)	103.7 ± 9.1	96.8 ± 12.2	<.001 (0.65)	.68
*Right leg reach*				
AT (% LL)	78.9 ± 9.5	71.5 ± 7.3	<.001 (0.87)	.74
PM (% LL)	119.5 ± 8.8	107.8 ± 12.1	<.001 (1.11)	.78
PL (% LL)	115.6 ± 9.1	104.2 ± 11.5	<.001 (1.10)	.78
CS (% LL)	104.6 ± 8.1	94.5 ± 9.4	<.001 (1.16)	.80
*Left leg reach*				
AT (% LL)	77.5 ± 9.2	70.3 ± 7.3	<.001 (0.86)	.74
PM (% LL)	118.5 ± 8.7	106.0 ± 12.3	<.001 (1.17)	.79
PL (% LL)	115.5 ± 10.6	103.2 ± 12.3	<.001 (1.08)	.77
CS (% LL)	103.8 ± 8.1	93.2 ± 9.6	<.001 (1.21)	.81

Data are mean ± standard deviation. Absolute values (cm) are shown first followed by relative values (% LL). Cohen’s *d* [18] can be classified as being small (0 ≤ *d* ≤ 0.49), medium (0.50 ≤ *d* ≤ 0.79), or large (*d* ≥ 0.80). In accordance with Deyo and Centor [23], the AUC-value can lie between 0.5 (“no” discriminative validity) and 1.0 (“perfect” discriminative validity)

*AT* anterior, *AUC* area under the receiver operator characteristic (ROC) curve, *CS* composite score, *LL* leg length, *PL* posterolateral, *PM* posteromedial

Concerning the relative values (i.e., reach distance in % LL), the soccer players achieved large-sized and significantly better values for AT (*p* < 0.001; *d* = 0.86–0.87), PM (*p* < 0.001; *d* = 1.11–1.17), and PL (*p* < 0.001; *d* = 1.08–1.10) directions as well as for the CS (*p* < 0.001; *d* = 1.16–1.21) compared to the controls, irrespective of the reaching leg. Additionally, we found significant differences between the three reach directions (PM > PL > AT) for the right and the left leg reach, irrespective of training status (i.e., soccer players or untrained subjects). Further, AUC-values were 0.74 (AT), 0.78 (PM), 0.78 (PL), and 0.80 (CS) for the right and 0.74 (AT), 0.79 (PM), 0.77 (PL), and 0.81 (CS) for the left leg reach, respectively. Results indicate that there is a chance of 74–81% that the YBT-LQ is possible to differentiate between soccer players and age-matched untrained individuals.

### Discriminative validity of upper quarter Y balance test performance (study 2)

Table 3 shows the statistics on the discriminative validity for YBT-UQ performance between young female and male swimmers and age−/sex-matched untrained subjects. With regard to the absolute values (i.e., reach distance in cm), we detected significant, large-sized differences in favour of swimmers for the MD (*p* < 0.001, *d* = 0.87–0.91), IL (*p* < 0.001, *d* = 1.19–1.30), and SL (*p* < 0.001, *d* = 1.46–1.78) directions. Further, we found significant differences between the three reach directions (MD > IL > SL) for the right and left arm reach, irrespective of training status (i.e., swimmers or untrained subjects). In addition, AUC-values were 0.75 (MD), 0.83 (IL), and 0.90 (SL) for the right and 0.75 (MD), 0.79 (IL), and 0.84 (SL) for the left arm reach, respectively. In other words, there is a chance of 75–90% that the YBT-UQ is possible to distinguish between swimmers and age−/sex-matched untrained subjects.

**Table 3:** Discriminative validity for the assessment of absolute (cm) and relative (% arm length) Upper Quarter Y Balance Test performance between trained (i.e., swimmers) and age−/sex-matched untrained (i.e., controls) youth

	Participants (*N* = 74)		Statistics	
	Swimmers (*n* = 37)	Controls (*n* = 37)	*p*-value (*d*)	AUC-value
*Right arm reach*				
MD (cm)	84.8 ± 8.8	76.3 ± 10.6	<.001 (0.87)	.75
IL (cm)	83.1 ± 10.7	69.7 ± 9.8	<.001 (1.30)	.83
SL (cm)	67.9 ± 8.3	53.6 ± 7.8	<.001 (1.78)	.90
*Left arm reach*				
MD (cm)	83.6 ± 8.9	75.1 ± 10.0	<.001 (0.91)	.75
IL (cm)	82.8 ± 9.7	70.7 ± 10.5	<.001 (1.19)	.79
SL (cm)	64.3 ± 7.3	52.6 ± 8.6	<.001 (1.46)	.84
*Right arm reach*				
MD (% AL)	104.0 ± 8.4	94.5 ± 11.9	<.001 (0.92)	.73
IL (% AL)	102.2 ± 13.5	87.2 ± 16.6	<.001 (0.99)	.76
SL (% AL)	83.3 ± 9.3	67.0 ± 12.6	<.001 (1.48)	.85
CS (% AL)	96.0 ± 8.1	82.9 ± 12.1	<.001 (1.28)	.80
*Left arm reach*				
MD (% AL)	102.3 ± 6.8	92.7 ± 10.4	<.001 (1.09)	.78
IL (% AL)	101.8 ± 12.9	88.3 ± 17.5	<.001 (0.88)	.71
SL (% AL)	79.0 ± 9.4	65.8 ± 13.8	<.001 (1.11)	.77
CS (% AL)	94.8 ± 9.0	82.3 ± 12.3	<.001 (1.17)	.79

Data are mean ± standard deviation. Absolute values (cm) are shown first followed by relative values (% AL). Cohen’s *d* [18] can be classified as being small (0 ≤ *d* ≤ 0.49), medium (0.50 ≤ *d* ≤ 0.79), or large (*d* ≥ 0.80). In accordance with Deyo and Conter [23], the AUC-value can lie between 0.5 (“no” discriminative validity) and 1.0 (“perfect” discriminative validity)

*AL* arm length, *AUC* area under the receiver operator characteristic (ROC) curve, *CS* composite score, *IL* inferolateral, *MD* medial, *SL* superolateral

With respect to the relative values (i.e., reach distance in % AL), we observed significant, large-sized differences in favour of swimmers for the MD (*p* < 0.001, *d* = 0.92–1.09), IL (*p* < 0.001, *d* = 0.88–0.99), and SL (*p* < 0.001, *d* = 1.11–1.48) directions as well as for the CS (*p* < 0.001, *d* = 1.17–1.28). In addition, AUC-values were 0.73 (MD), 0.76 (IL), 0.85 (SL), and 0.80 (CS) for the right and 0.78 (MD), 0.71 (IL), 0.77 (SL), and 0.79 (CS) for the left arm reach, respectively. In other words, there is a chance of 71–85% that the YBT-UQ is possible to distinguish between swimmers and age−/sex-matched untrained subjects.

## Discussion

To our knowledge, the present studies investigated discriminative validity of YBT-LQ/UQ performance between healthy trained and untrained youth for the first time. Main results for the relative/normalized measures (i.e., reach distance in % LL/AL) can be summarized as follows and were confirmed by the same analyses using the absolute/raw measures (i.e., reach distance in cm): (1) trained youth (i.e., soccer players and swimmers) showed large-sized significantly better YBT-LQ/UQ performance compared with age−/sex-matched untrained controls; (2) ROC analyses revealed a chance of ≥74% (YBT-LQ) and ≥ 71% (YBT-UQ) to discriminate youth athletes from untrained youth.

In line with our hypothesis stating better performances in both tests for trained compared to untrained youth, one-way ANOVA revealed significantly larger absolute (cm) and relative (% LL/AL) YBT-LQ/UQ reach distances in trained participants (i.e., soccer players and swimmers) compared to age−/sex-matched controls. This result corresponds with findings from studies [3, 13–15, 24, 25] that investigated groups of athletes with varying levels of competition (e.g., high school vs. collegiate vs. professional players) and reported better relative YBT-LQ/UQ performance for those with a higher than for those with a lower competition level. However, besides the differences in competition level, the included persons also differed in age (i.e., adults versus adolescents), which might have influenced the results. In the present study, we included age−/sex-matched controls and results nonetheless showed better absolute and relative YBT-LQ/UQ-performance in trained compared to untrained youth. This finding is in line with a study by Engquist et al. [26] that investigated young adults (mean age: 20 ± 1.6 years) and found larger YBT-LQ reach distances in trained (i.e., female Division I student-athletes) compared to non-trained but same-aged (i.e., general female college students) individuals. Our findings and the results by Engquist and colleagues indicate that differences in absolute and relative YBT-LQ/UQ performance in age- and sex-matched individuals are based on training status and competition level. Additionally and also in accordance with our hypothesis stating good discriminative validity for the YBT-LQ/UQ in healthy youth, our ROC analysis for the relative data yielded a chance between 74 to 81% (YBT-LQ) and 71 to 85% (YBT-UQ) to discriminate youth athletes from age−/sex-matched untrained youth and this was confirmed using the absolute data. Thus, YBT-LQ (i.e., dynamic balance) and YBT-UQ (i.e., shoulder mobility/stability) demands seem to be associated with respective athletic requirements in soccer and swimming.

What might be the reason for performance differences in YBT-LQ/UQ between trained and age−/sex-matched untrained youth? One might argue that trained compared to non-trained youth possess a higher amount of long-lasting, continuous and intense training experience [27]. Another reason might be the genetic profile of trained individuals [28, 29]. For example, Murtagh et al. [28] investigated the relationship of multiple single nucleotide polymorphisms with physical performance measures in elite male youth soccer players and control participants. The authors observed differences in the genetic profile (e.g., higher genotype frequency distribution in soccer players) and showed that physical performance was associated with some measures of the genetic profile. Taken together, both preconditions cause specific adaptations [30, 31] that allow for higher performances in sport-specific as well as in physical fitness measures.

From a practical perspective, our findings of significantly better absolute and relative YBT-LQ/UQ values in trained compared to untrained youth and the good discriminative validity indicate that both tests can be used to distinguish between young athletes and age−/sex-matched controls based on dynamic balance (YBT-LQ) and shoulder mobility/stability (YBT-UQ) data. Consequently, both testing procedures can be used to discriminate persons with higher compared to lower levels of performance. This allows for the possibility to offer specifically tailored sport programs to support growing-ups according to their individual performance level, e.g., fitness promoting programs for low fit individuals and young athlete training regimens for high fit subjects.

Of note, our findings are limited to two cohorts (i.e., soccer players and swimmers). Both groups represent cohorts that are used to control their postural stability in challenging situations (i.e., soccer players) or to maintain mobility/stability in their pectoral girdle and upper extremities (i.e., swimmers). Consequently, our findings cannot be generalized to other populations or sports and further research is needed to examine athletes from different disciplines or other cohorts.

## Conclusions

We investigated the discriminative validity of absolute and relative YBT-LQ/UQ performance between healthy trained (i.e., soccer players and swimmers) and untrained youth (i.e., age−/sex-matched controls) and found significantly better values for the former one as well as good discriminative validity for both tests. Our findings indicate that both, the YBT-LQ and the YBT-UQ are suitable field tests to effectively differentiate between trained and age−/sex-matched untrained youth based on dynamic balance (YBT-LQ) and shoulder mobility/stability (YBT-UQ) data.

## Data Availability

The data generated and analyzed during the present study are not publicly available due to ethical restrictions but are available from the corresponding author upon reasonable request.

## Abbreviations

AL: Arm length
ANOVA: Analysis of variance
AT: Anterior
AUC: Area under the receiver operator characteristic curve
BMI: Body mass index
CS: Composite score
IL: Inferolateral
LL: Leg length
MD: Medial
PHV: Peak height velocity
PL: Posterolateral
PM: Posteromedial
ROC: Receiver operator characteristic curve
SL: Superolateral
YBT-LQ: Lower Quarter Y Balance Test
YBT-UQ: Upper Quarter Y Balance Test
